# Fluorescence Microscopy-Based Quantitation of GLUT4 Translocation: High Throughput or High Content?

**DOI:** 10.3390/ijms21217964

**Published:** 2020-10-27

**Authors:** Verena Stadlbauer, Peter Lanzerstorfer, Cathrina Neuhauser, Florian Weber, Flora Stübl, Petra Weber, Michael Wagner, Birgit Plochberger, Stefan Wieser, Herbert Schneckenburger, Julian Weghuber

**Affiliations:** 1 School of Engineering, University of Applied Sciences Upper Austria, Stelzhamerstraße 23, 4600 Wels, Austria; cathrina.neuhauser@ffoqsi.at (C.N.); flora.stuebl@fh-wels.at (F.S.); 2 FFoQSI GmbH-Austrian Competence Centre for Feed and Food Quality, Safety and Innovation, Technopark 1C, 3430 Tulln, Austria; 3 School of Medical Engineering and Applied Social Sciences, University of Applied Sciences Upper Austria, Garnisonstraße 21, 4020 Linz, Austria; florian.weber2@fh-linz.at (F.W.); birgit.plochberger@fh-linz.at (B.P.); 4 Institute of Applied Research, Aalen University, Beethovenstraße 1, 73430 Aalen, Germany; petra.weber22@web.de (P.W.); michael.wagner@hs-aalen.de (M.W.); herbert.schneckenburger@hs-aalen.de (H.S.); 5 ICFO-Institut de Ciencies Fotoniques, The Barcelona Institute of Science and Technology, 08860 Castelldefels, Spain; stefan.wieser@icfo.eu

**Keywords:** GLUT4 translocation, TIRF microscopy, TIR multiwell reader, GPMV formation, diabetes mellitus, insulin mimetic compounds

## Abstract

Due to the global rise of type 2 diabetes mellitus (T2DM) in combination with insulin resistance, novel compounds to efficiently treat this pandemic disease are needed. Screening for compounds that induce the translocation of glucose transporter 4 (GLUT4) from the intracellular compartments to the plasma membrane in insulin-sensitive tissues is an innovative strategy. Here, we compared the applicability of three fluorescence microscopy-based assays optimized for the quantitation of GLUT4 translocation in simple cell systems. An objective-type scanning total internal reflection fluorescence (TIRF) microscopy approach was shown to have high sensitivity but only moderate throughput. Therefore, we implemented a prism-type TIR reader for the simultaneous analysis of large cell populations grown in adapted microtiter plates. This approach was found to be high throughput and have sufficient sensitivity for the characterization of insulin mimetic compounds in live cells. Finally, we applied confocal microscopy to giant plasma membrane vesicles (GPMVs) formed from GLUT4-expressing cells. While this assay has only limited throughput, it offers the advantage of being less sensitive to insulin mimetic compounds with high autofluorescence. In summary, the combined implementation of different fluorescence microscopy-based approaches enables the quantitation of GLUT4 translocation with high throughput and high content.

## 1. Introduction

Diabetes mellitus (DM) is a metabolic disease that leads to high blood glucose levels in the body over a prolonged period. This condition is caused either by pancreatic disorders resulting in low insulin levels (type 1 diabetes mellitus, T1DM) or insulin-resistant cells that do not respond properly to insulin (type 2 diabetes mellitus, T2DM). Generally, insulin increases the uptake, utilization, and storage of glucose by muscle and adipose tissue. In addition, it inhibits endogenous glucose production by the liver [1]. Approximately three-quarters of postprandially elevated blood glucose is decreased by muscle tissue [2]. The effect of insulin is initiated by binding to its receptor, leading to an increase in receptor tyrosine kinase activity and downstream tyrosine phosphorylation of insulin receptor substrate-1 (IRS-1), and activation of phosphatidylinositol-3 kinase (PI3K) and protein kinase B/Akt. Finally, this cascade promotes the exocytic trafficking of intracellular membranes containing glucose transporter 4 (GLUT4) proteins to enable a fast increase in glucose uptake [3,4,5,6].

The International Diabetes Federation (IDF) has recently reported that T2DM is currently increasing. Approximately 390 million people were affected, and approximately 4.5 million deaths were reported in 2019 [7]. Importantly, T2DM is associated not only with hyperglycemia and insulin resistance but also with obesity, hypertension, and hyperlipidemia. This combination is known as metabolic syndrome and is a high-risk factor for cardiovascular diseases, resulting in many further deaths [8]. Untreated T2DM leads to damage to various tissues, including the liver, nerves, kidney, and brain [9]. The available oral T2DM treatment strategies exhibit limitations due to low drug efficacy over time and side effects, such as diarrhea, vomiting, cramps, increased risk of heart failure, pancreatitis, or ketoacidosis [10,11,12]. Therefore, more effective synthetic or natural compounds to treat insulin resistance are urgently needed.

A promising strategy to obtain such insulin mimetic drugs is the application of screening approaches that are based on the analysis of GLUT4 translocation. This analysis is conventionally performed by immunostaining GLUT4 containing an epitope tag in the exofacial loop [13]. Unfortunately, many wet-lab methods, including Western blot analysis of membrane fractions, ELISA-guided binding assays, and flow cytometric analysis [14,15,16], are indirect and therefore prone to errors, are time consuming, are nonquantitative, and have a low sensitivity. Therefore, there is an urgent need for alternative approaches that are robust, high throughput, and sensitive. A luminescence-based system described previously could be an appropriate solution in this regard [17]. However, the sensitivity and ease of implementation in comparison to fluorescence-based assays might be lower.

Fluorescence microscopy has been widely used for the analysis of GLUT4 translocation, with confocal microscopy being the most popular technique [13,18]. Furthermore, total internal reflection fluorescence (TIRF) microscopy has been used to investigate GLUT4 trafficking [19], also at high resolution [20,21,22]. Objective-type TIRF using a microscope objective lens with a very high numerical aperture has been demonstrated to be a good approach, but it still has limited throughput [23]. Prism-type TIRF with light incidence via a glass or quartz prism whose refractive index exceeds that of the specimen offers advantages in regard to throughput but with lower resolution [24]. Prism-type TIRF has been successfully implemented to perform simultaneous measurements in 96-well microtiter plates using a device termed the “TIR reader” [25,26].

An important disadvantage of confocal microscopy is the high background of intracellular fluorophores. This problem can be addressed by the formation of giant plasma membrane vesicles (GPMVs) that are isolated directly from living cells and contain plasma membrane-derived GLUT4 molecules [27]. The preparation of these vesicles is simple, and their analysis can be performed by confocal or epifluorescence microscopy.

In this study, we report on the efficacy of three different microscopic approaches for the quantitation of GLUT4 translocation: (1) objective-type scanning TIRF microscopy, (2) a prism-type TIR reader for the simultaneous analysis of large cell populations grown in microtiter plates, and (3) confocal microscopy of GPMVs obtained from GLUT4-GFP-expressing cells. We present their particular efficacy based on the application of insulin and insulin mimetics, plant-derived compounds, and discuss their pros and cons in terms of sensitivity, handling, and throughput.

## 2. Results

### 2.1. Quantitation of GLUT4 Translocation by Objective-type Scanning TIRF Microscopy

In the first approach, we quantitated the translocation of GLUT4 in a large number of CHO-K1 [19] and HeLa cells (this study) stably expressing the GLUT4-myc-GFP fusion protein. For our experiments, we used a microscopy system in an objective-type TIRF configuration combined with a focus-hold system and a motorized scanning table. This setup allows the fast imaging of thousands of cells within a short time by automatically scanning multiple wells of a microtiter plate. Software developed by our research group [28] further enabled a fast, semi-automated calculation of the fluorescent signal to shorten the required experiment time.

**Figure 1:**
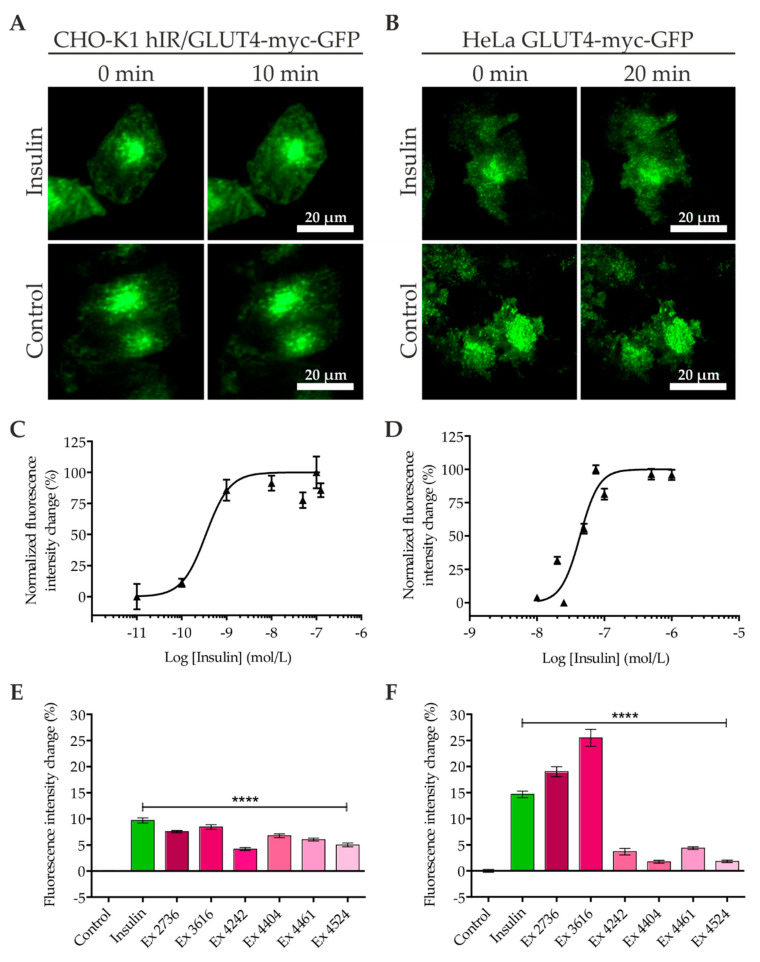
Glucose transporter 4 (GLUT4) translocation quantitation by objective-type scanning total internal reflection fluorescence (TIRF) microscopy. CHO-K1 (**A**,**C**,**E**) and HeLa (**B**,**D**,**F**) cells stably expressing a GLUT4-myc-GFP fusion protein were seeded in 96-well microtiter plates and starved for 3 h in HBSS buffer on the following day. Images in the TIRF configuration were taken before and after stimulation with insulin for 10 (**A**) or 20 min (**B**). Insulin dose–response curves were generated by quantitation of the fluorescence signal intensity increase induced by various insulin concentrations in both cell lines (**C**,**D**). Six different plant extracts were tested for their insulin mimetic properties (**E**,**F**). Values represent the mean ± SEM (*n* > 500). **** *p <* 0.0001 indicates statistically significant differences from the untreated controls.

The cells were grown in TIRF-capable 96-well microtiter plates, starved, and incubated with insulin diluted in Krebs–Ringer phosphate–HEPES (KRPH) buffer. Subsequently, the increase in the fluorescence intensity in the evanescent field, which correlates with an elevated GLUT4 translocation [19,29], was determined. As indicated in Figure 1A,B, we analyzed the fluorescence in the same cells before and after the addition of insulin and quantitated the fluorescence signal of approximately 500 cells for each concentration. As expected, we found an insulin concentration-dependent increase in the GLUT4-GFP signal, that is, incubation with various insulin concentrations for 10 (CHO-K1 cells) or 20 (HeLa cells) minutes resulted in a characteristic dose–response relationship with a calculated EC50 value of 0.35 nM for CHO-K1 cells (Figure 1C). Figure 1D indicates a lower responsiveness of HeLa cells with a calculated EC50 value of 44 nM. Furthermore, we quantitated the GLUT4-GFP signal at different time points after insulin stimulation. While we found a full response in CHO-K1 cells after 10 min, the signal in HeLa cells continued to increase up to 30 min. The temporal resolution of GLUT4 translocation is shown in Figure S1.

Both cell lines were also used to study the efficacy of various plant extracts in inducing GLUT4 translocation. Six different extracts with known activity obtained from a commercial library (PECKISH) [30] were chosen for this study. These extracts were recently identified in a large-scale screening approach that was performed in our laboratory. As shown in Figure 1E,F, two extracts (#2736 and #3616) resulted in a strong response comparable to or even higher than insulin if applied at low concentrations (1 mg/L). The other four tested extracts (#4242, #4404, #4461, and #4524) led to a minor (HeLa) or moderate (CHO-K1) effect. The temporal resolution of the extracts is indicated in Figure S1C,D. In conclusion, objective-type scanning TIRF microscopy represents an approach with an extremely high sensitivity and moderate throughput for the identification and qualification of insulin mimetic compounds, confirming the results of our previous studies [19,29,31].

### 2.2. Quantitation of GLUT4 Translocation Using A Prism-Type TIR Reader

As described previously, objective-type TIRF microscopy is a highly sensitive method for the quantitation of GLUT4 translocation. However, throughput is still limited, and this technique is not applicable for the screening of thousands or tens of thousands of compounds. In search of alternative methods, we tested an approach based on prism-type TIRF. This system, termed the “TIR reader”, was previously developed and implemented in our laboratory and allows simultaneous analysis of large cell populations grown in microtiter plates [25,26].

**Figure 2:**
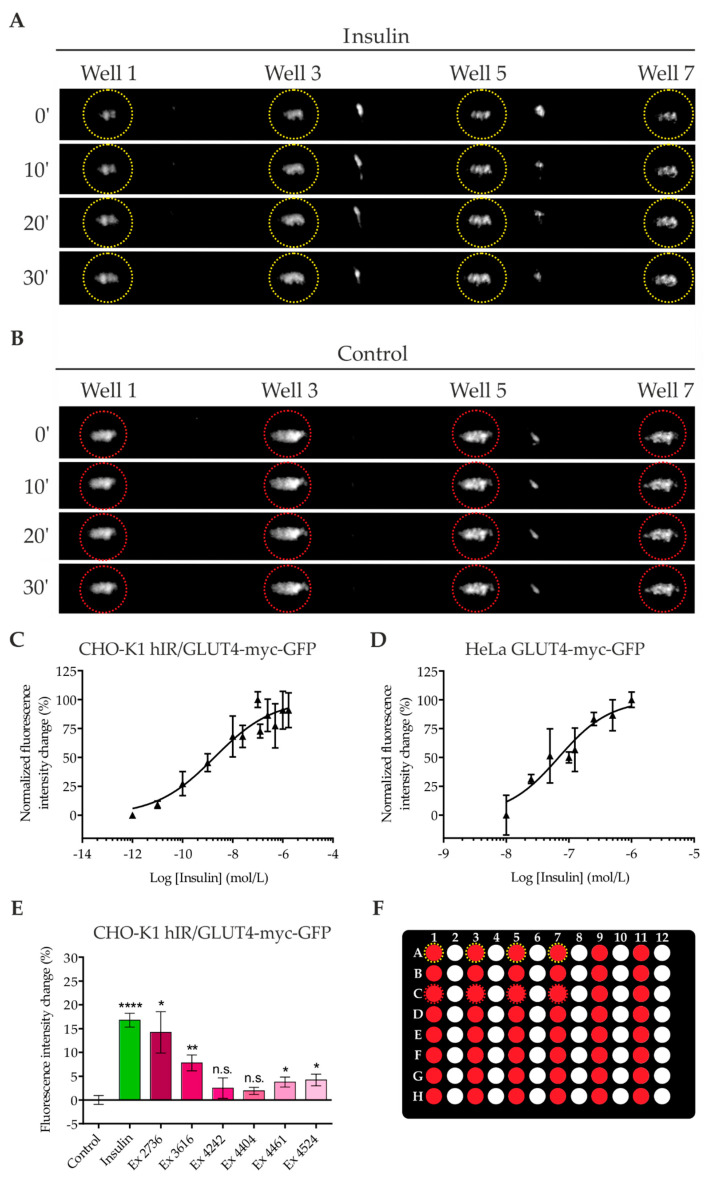
Quantitation of GLUT4 translocation using a prism-type TIR reader. CHO-K1 (**A**–**C**,**E**) and HeLa (**D**) cells stably expressing a GLUT4-myc-GFP fusion protein were seeded into adapted 96-well microtiter plates and starved for 3 h in HBSS buffer on the following day. Images in the TIR configuration were taken before and after stimulation with insulin or the indicated plant extracts. Four selected wells with insulin-treated (yellow dashed circles, (**A**) and (**F**)) or control cells (red dashed circles, (**B**) and (**F**)) were used for illustration. Insulin dose–response curves were generated by quantitation of the fluorescence signal intensity increase induced within 10 (CHO-K1) and 20 (HeLa) min by various insulin concentrations (**C**,**D**). Values represent the mean ± SEM (*n* > 500). * *p* < 0.05, ** *p* < 0.01, and **** *p* < 0.0001 indicate statistically significant differences from the untreated control.

To compare the approach with objective-type TIRF, we used the same CHO-K1 and HeLa cell lines that express GLUT4-myc-GFP. Cells were seeded in specialized microtiter plates with an appropriate glass bottom (2 mm thickness; machined and polished border), starved, and stimulated with insulin at different concentrations. As shown in Figure 2A,B, stimulation with 100 nM insulin resulted in a strong increase in fluorescence in a time-dependent manner. For our measurements, we selected four different wells spread across the microtiter plate, in this case rows A and C and columns 1, 3, 5, and 7 (Figure 2F). A detailed description of the experimental setup, including the readout, is depicted in Figure S2 (Supplementary Materials). Experiments were then performed at different insulin concentrations, resulting in a dose–response relationship with a calculated EC50 value of 1.9 nM for CHO-K1 cells and 69 nM for HeLa cells (Figure 2C,D). A temporal resolution of GLUT4 translocation in both cell lines is shown in Supplementary Figure S3A,B.

Additionally, we tested the same six aforementioned PECKISH-derived plant extracts at a concentration of 1 mg/L in CHO-K1 cells. As indicated in Figure 2E, incubation with extracts #2736 and #3616 for 10 min led to a strong increase in the GFP signal, comparable or slightly lower than that of the insulin control. The other four tested extracts showed no significant or only a minor increase. The temporal resolution of the extracts is indicated in Figure S1C. In conclusion, the results from experiments performed with the TIR reader are in good agreement with those obtained from objective-type TIRF.

### 2.3. Influence of Autofluorescence of Tested Compounds on the Quantitation of GLUT4 Translocation

**Figure 3:**
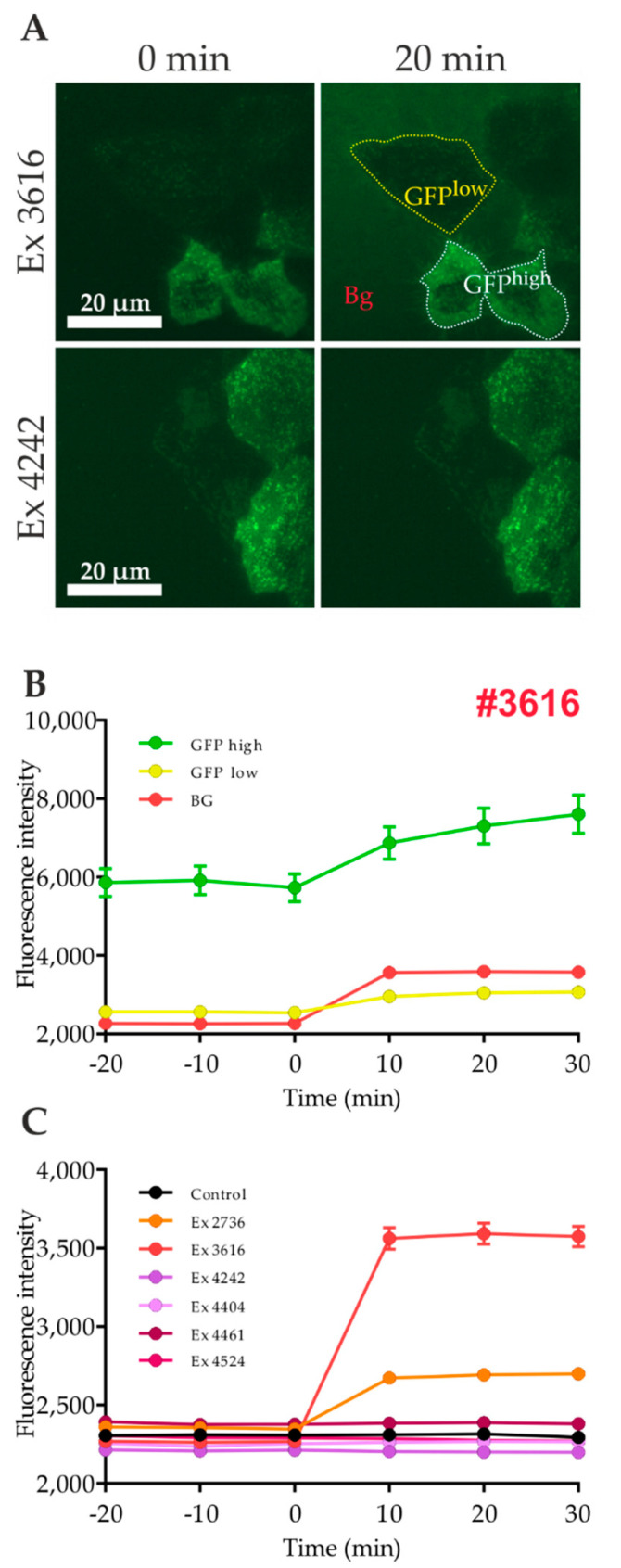
Analysis of the potential autofluorescence of the tested plant extracts by objective-type TIRF microscopy. HeLa cells stably expressing a GLUT4-myc-GFP fusion protein were seeded in 96-well microtiter plates and starved for 3 h in HBSS buffer on the following day. Images in the TIR configuration were taken before and after stimulation with the indicated extract for 10, 20, and 30 min. (**A**) The fluorescence signal intensity of cell-free regions (background, bg) and regions with cells expressing high or low levels of GLUT4-myc-GFP (GFP^high^, GFP^low^) were separately analyzed. The results of a representative extract (#3616) with strong autofluorescence are shown in (**B**). All plant extracts under study were tested for their background fluorescence intensities (**C**). Values represent the mean ± SEM (*n* > 200).

While performing the screening assay based on objective-type TIRF microscopy, we realized that a considerable number of plant-derived extracts exhibited autofluorescence at the chosen excitation wavelength. Naturally, this phenomenon may lead to false positive hits. Therefore, we studied this topic in more detail and retested the extracts described in Section 2.1. As shown in Figure 3A, application of extract #3616, which was classified as a putative autofluorescent extract when performing the screening run, resulted in a significant increase in the fluorescence signal in cell-free regions (termed “background, bg”). However, in cell-positive regions, the degree of the fluorescence intensity increase was different, depending on the basal GFP expression level (termed “GFP high” and “GFP low”; Figure 3B). Most of the other tested extracts, such as #4242, did not lead to an increase in the background level (Figure 3A,C). Based on these results, we concluded that an increase in the GFP signal in cell-positive regions (which are solely relevant for the quantitation procedure) cannot be caused only by the autofluorescence of the applied compound. However, a minor impact cannot be excluded.

Therefore, we recorded fluorescence spectra of the plant extracts at three concentrations (1, 10, and 25 mg/L) within the excitation range of 450 to 530 nm (Figure S4A). Results show a significant increase with 10 or 25 mg/L, especially for the extracts #2736 and #3616. These findings are in good agreement with results obtained from objective-type TIRF measurements. Furthermore, quenching properties of the plant extracts were tested by the addition of soluble GFP (Figure S4B). Here, the extracts #2736 and #3616 caused a strong decrease in the GFP signal already at 1 mg/L. Thus, autofluorescence and quenching properties of test substances are potential factors that need to be considered in fluorescence microscopy-based GLUT4 translocation experiments.

### 2.4. Quantitation of GLUT4 Translocation by Confocal Microscopy of Giant Plasma Membrane Vesicles (GPMVs)

**Figure 4:**
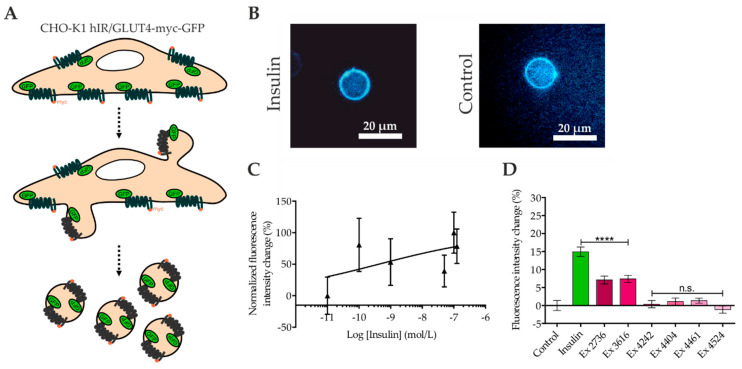
Quantitation of GLUT4 translocation by confocal microscopy of giant plasma membrane vesicles (GPMVs) prepared from CHO-K1 cells stably expressing a GLUT4-myc-GFP fusion protein. These cells were seeded in 96-well microtiter plates, starved for 3 h in HBSS buffer on the following day, and incubated with the test substances for 10 min. The cells were washed three times with GPMV buffer and incubated for 1 h in GPMV-forming buffer. The GPMVs were transferred to empty wells of an imaging plate, and images were taken by confocal microscopy. A schematic illustration of GPMV formation (**A**) and formed GPMVs of the insulin-treated and untreated cells (**B**) is shown. The insulin dose–response curve was generated by quantitation of the fluorescence signal intensity increase induced within 10 min by various insulin concentrations (**C**). Fluorescence intensities of the GPMVs from the cells treated with insulin and the indicated plant extracts were analyzed and normalized to those of the untreated cells (**D**). Values represent the mean ± SEM (*n* > 1000). **** *p* < 0.0001 indicates statistically significant differences from the untreated controls. n.s., not significant.

GPMVs are isolated directly from living cells and represent an interesting experimental model system for use in a wide range of approaches. They capture much of the compositional protein and lipid complexity of intact cell plasma membranes, are filled with cytoplasm, and are free from contamination with membranes from internal organelles [32]. As GPMVs are formed only after treatment with the compound of interest and then collected from the incubation buffer for further analysis (Figure 4A), we speculated that these membrane vesicles might be of interest for analysis of plasma membrane-localized GLUT4 molecules. As a major advantage, the potential autofluorescence of the applied compound should not be relevant due to several washing steps and the transfer of formed GPMVs into a fresh microtiter plate for the final analysis. For implementation, CHO-K1 GLUT4-myc-GFP cells were grown in 96-well microtiter plates, starved, and incubated with insulin or the plant extracts described before dilution in KRPH buffer. Subsequently, formed GPMVs were transferred to appropriate microtiter plates and analyzed by confocal microscopy (Figure 4B). For a dose–response relationship, several insulin concentrations were analyzed and the results indicate a maximum response already at 0.1 nM insulin with an EC50 value of 0.42 nM (Figure 4C). As indicated in Figure 4D, treatment with 100 nM insulin resulted in a significant increase in the fluorescence intensity compared to that of the control GPMVs. Furthermore, the PECKISH extracts #2736 and #3616 significantly increased the number of GLUT4 molecules, whereas the other four tested extracts resulted in only minor changes in the fluorescence intensity. In conclusion, the confocal microscopy findings of the GPMVs were consistent with the results obtained from the TIRF measurements.

## 3. Discussion

Given the increasing prevalence of lifestyle diseases, including metabolic syndrome, the treatment of T2DM and insulin resistance [33] remains a key topic of both academic [34] and traditional medicine [35]. An important strategy in the search for antidiabetic compounds is the improvement of glucose uptake in adipose and muscle tissue. Therefore, insulin mimetic compounds that induce translocation of GLUT4 molecules into the plasma membrane in the absence of insulin are important.

In recent years, numerous assays have been developed to address this question, with fluorescence microscopy as a key technology in this context. The application of fluorescent glucose analogs such as 2-NBDG [36] is challenging, and a precise evaluation of uptake into living cells using such strategies is difficult in many cell lines [37]. The reasons include low membrane permeability and weak intensity of the applied tracing molecules. While there has been good progress in the development of novel glucose analogs in recent years [38], fluorescent GLUT4 fusion proteins remain advantageous in terms of cost, handling, and application potential.

In this study, we investigated and compared three different microscopy techniques (objective-type TIRF, prism-type TIRF, and confocal microscopy) applied to live cells and giant plasma membrane vesicles (GPMVs) for their suitability, sensitivity, and throughput for a quantitative analysis of GLUT4 translocation. Therefore, we used two different cell lines stably expressing GLUT4-myc-GFP, namely, CHO-K1, as previously described [19], and HeLa, established for this study. Both cell lines grow fast and can be used without the need for differentiation, which was challenging and time-consuming in previous studies [29]. In a recent study, it was demonstrated that HeLa cells expressing a similar GLUT4 fusion protein (HA-GLUT4-GFP) show comparable kinetics and orthologous trafficking mechanisms to 3T3-L1 adipocytes [39], confirming their applicability for our approach. In addition, these cells exhibit good adhesion properties on various surfaces that have been implemented for microscopy, which is especially important for TIRF microscopy. We found a ~100-fold higher sensitivity of CHO-K1 cells, which is possibly caused by the high expression of the human insulin receptor in this cell line as demonstrated previously [19].

**Figure 5:**
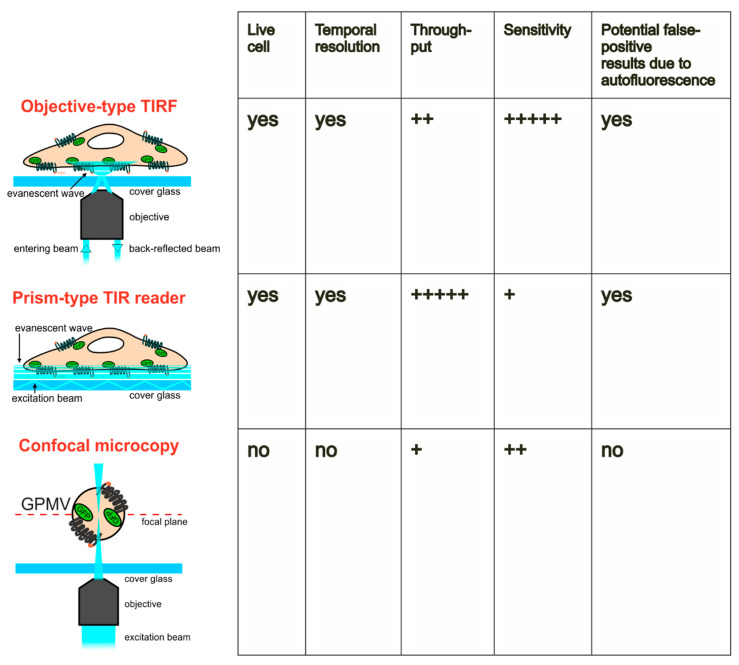
Overview of three fluorescence microscopy-based methods for the quantitation of GLUT4 translocation in cells or giant plasma membrane vesicles (GPMVs). Pros and cons of objective-type TIRF microscopy, a prism-type TIR reader, and confocal microscopy of GPMVs are indicated and rated. Schematic illustrations are simplified and not drawn to scale. +: very low; ‚++: low; +++++: very high.

As shown in several studies in recent years, objective-type TIRF was approved as a powerful tool for the quantitation of GLUT4 translocation. This technique has extremely high sensitivity and allows analysis of GLUT4 translocation in live cells with temporal resolution (see Figure 5 for a comparison of the methods presented in this study). The setups used in our labs are equipped with scanning stages of high precision, and quantitation is performed by semi-automated software including cell detection and other features for an optimized analysis [28]. Thus, the throughput of objective-type TIRF was significantly increased for this application, enabling the screening of hundreds of plant extracts and other synthetic and natural compounds within several months. This strategy led to the identification of compounds of interest for application in nutraceuticals and food supplements [19,29,31,40].

However, we did not achieve our original goal, which was the screening of thousands of compounds and extracts, such as the PECKISH library [30], in the desired time. Usage of the current objective-type TIRF system allows for the analysis of only ~12 wells in 4–5 min. Further upscaling is difficult due to technical limitations, especially the interruptions of the oil film of the objective upon stage movement. For this reason, we tested a prism-type TIR reader, which we previously used to quantitate protein–protein interactions [25,26]. This system allows simultaneous quantitation of the fluorescence intensity in live cells grown in 96-well microtiter plates. By adjusting the thickness of the glass bottom, we could also use 384-well plates, which would further increase the throughput. The obtained results clearly showed that the TIR reader enables the quantitation of GLUT4 translocation with sufficient sensitivity, that is, insulin as well as selected natural insulin mimetic compounds led to a significant increase in the GLUT4-GFP signal in the evanescent field. This effect was confirmed in both applied cell lines and, for insulin, in a dose-dependent manner. Consistent with the experiments performed in objective-type TIRF, we detected a similar increase in the range of ~10–15% for insulin and two effective plant extracts (#2736 and #3613). Taken together, the results obtained from objective-type and prism-type TIRF were well correlated, confirming the suitability of both approaches.

As both TIRF-based approaches are highly sensitive, potential autofluorescence of applied plant extracts may falsify the results. To address this question, an additional pre-screening experiment to unravel autofluorescent extracts was performed. Therefore, we recorded fluorescence spectra in the relevant excitation range between 450 and 530 nm. In fact, some extracts resulted in significant fluorescent signals, but only at elevated concentrations of 10 and 25 mg/L. In conclusion, a direct comparison of results obtained from GLUT4 TIRF measurements and spectrometric extract analysis may not be appropriate. However, this measurement represents a good tool to get first hints on highly autofluorescent substances before starting GLUT4 analysis. Additionally, we investigated potential GFP quenching properties of the plant extracts. We found that some plant extracts lead to decreased fluorescence intensities of soluble GFP. As the GFP of the GLUT4 fusion protein is localized to the intracellular region and TIRF microscopy experiments excluded high membrane permeability of the extracts, a quenching effect in live-cell experiments appears negligible. Furthermore, a strong quenching effect would only result in false negatives or underestimated bioefficacy of the test compound.

Finally, we generated giant plasma membrane vesicles (GPMVs) from CHO-K1 GLUT4-myc-GFP cells that were pretreated with insulin or selected plant extracts. These cells led to the efficient formation of GPMVs, while HeLa cells appeared to be less appropriate and were therefore not used for further analysis. Confocal microscopy was then used to quantitate the GFP signal in the membrane of the GPMVs. This approach is of special interest. First, the potential autofluorescence of the applied compounds is not relevant. After treatment with the effective compound, cells are washed several times in the course of GPMV formation and are finally transferred into new microtiter plates for microscopy experiments. Second, GPMVs are formed from the whole plasma membrane, while TIRF measurements are restricted to the basal membrane of the used cells (Figure 4). Data generated by quantitation of the GFP signal in the analyzed GPMVs were found to be consistent with those obtained from both applied TIRF approaches. Thus, confocal microscopy of GPMVs represents a good extension to whole-cell analysis performed by TIRF microscopy. The throughput of the approach in its used configuration is rather low, limiting its applicability as a screening tool. This limitation could be overcome by the application of confocal systems optimized for screening assays [41]. However, the GPMV formation procedure remains a limiting factor in this regard.

In summary, we implemented and validated three microscopy-based approaches for the quantitation of GLUT4 translocation. Potentially, these methods could also be used for translocation analysis of other proteins, including the GLUT4 trafficking regulator TUSC5 [42], the signal transduction adaptor protein Grb2 [43,44], or other transport proteins, such as the fatty acid translocase CD36 [45]. Figure 5 summarizes the main methodological features as well as the pros and cons. In short, the GPMV system offers the main advantage of being non-susceptible to autofluorescence caused by the applied compound. However, it lacks the possibility of live-cell analysis. In addition, a temporal resolution of the translocation process in a single GPMV cannot be achieved, that is, GPMVs are prepared at a defined time point, excluding the possibility to measure the same GPMV at multiple incubation times with the respective substance. Both options are available for objective-type and prism-type TIRF. The main differences of the TIRF assays are throughput, which is much better for prism-type TIRF, and sensitivity, which is higher for objective-type TIRF, as it is based on the analysis of single cells.

## 4. Materials and Methods

### 4.1. Reagents

Human insulin, HEPES, CaCl_2_, NaCl, KCl, MgSO_4_, KH_2_PO_4_, paraformaldehyde (PFA), and dithiothreitol (DTT) were purchased from Sigma-Aldrich (Schnelldorf, Germany). Phosphate buffered saline (PBS), Hank’s balanced salt solution (HBSS), Ham’s F12 culture medium, and RPMI 1640 culture medium were obtained from PAN-Biotech (Aidenbach, Germany). For preparation of test solutions, the plant extracts were dissolved in Krebs–Ringer phosphate–HEPES buffer (KRPH; 20 mmol/L HEPES, 1 mmol/L CaCl_2_, 136 mmol/L NaCl, 4.7 mmol/L KCl, 1 mmol/L MgSO_4_, and 5 mmol/L KH_2_PO_4_, pH 7.4). A library containing 2300 water-soluble plant extracts (PECKISH) [30] was provided by Frank Döring (Christian-Albrechts University, Kiel, Germany). For analysis of quenching properties, *Aequorea victoria* GFP, recombinant (His Tag) was purchased from Biomedica Medizinprodukte (Vienna, Austria). For GPMV formation, GPMV buffer (10 mmol/L HEPES, 2 mmol/L CaCl_2_, and 150 mmol/L NaCl, pH 7.4) and GPMV-forming buffer (25 mmol/L PFA, 2 mmol/L DTT, 10 mmol/L HEPES, 2 mmol/L CaCl_2_, and 150 mmol/L NaCl, pH 7.4) were used.

### 4.2. Cell Culture and Transfection

All cells were grown in a humidified atmosphere (≥95%) at 37 °C and 5% CO_2_. CHO-K1 cells stably expressing hIR and GLUT4-myc-GFP [19,46] were a kind gift from Manoj K. Bhat (National Centre for Cell Science, University of Pune, Pune, India). A fluorescence-activated cell sorter was used to further increase the number of positive cells, which were maintained in Ham’s F12 culture medium supplemented with 100 μg/mL penicillin, 100 μg/mL streptomycin, 1% G418, and 10% fetal bovine serum (FBS) (all from PAN-Biotech, Aidenbach, Germany). HeLa cells were obtained from ATCC (Manassas, VA, USA) and cultivated in RPMI 1640 medium supplemented with 100 μg/mL penicillin, 100 μg/mL streptomycin, and 10% FBS. For the generation of stable clones, cells were transfected with 1–5 μg DNA at 50–70% confluence using Lipofectamine LTX Reagent with PLUS Reagent (Thermo Fisher Scientific, Vienna, Austria) according to the manufacturer’s instructions. Cells were plated into 60 mm culture dishes and grown for 48 h. The medium was removed and replaced by medium supplemented with 400 μg/mL G418. This medium was changed every 3 days, and 15–20 days later; individual neomycin-resistant colonies were selected for propagation and analysis. For subsequent cell maintenance, 1% G418 was added to the growth medium.

### 4.3. Objective-Type TIRF Microscopy

CHO-K1 hIR/GLUT4-myc-GFP or HeLa GLUT4-myc-GFP cells were grown in 96-well imaging plates (45,000 cells/well; MoBiTec, Goettingen, Germany) overnight. After the cells were washed twice with HBSS, they were starved with HBSS for 3 h. The cells were incubated with human insulin or plant extracts dissolved in KRPH or KRPH alone as a control. Images were recorded up to 30 min in a 10 min interval. Two TIRF microcopy systems were used for imaging as follows. (i) An epi-fluorescence microscope (Nikon Eclipse Ti2, Tokyo, Japan) coupled with a multilaser engine (SLE; Toptica Photonics, Munich, Germany) was used for selective fluorescence excitation of GFP at 488 nm. The samples were illuminated in a total internal reflection (TIR) configuration (Nikon Ti-LAPP, Tokyo, Japan) using a 60× oil immersion objective (NA = 1.49, APON 60XO TIRF). The samples were mounted on an x-y-stage (CMR-STG-MHIX2-motorized table, Märzhäuser, Wetzlar, Germany), and scanning of the larger areas was supported by a laser-guided automated Perfect Focus System (Nikon PFS, Tokyo, Japan). After appropriate filtering, the fluorescence was imaged onto an sCMOS camera (Zyla 4.2, Andor, Belfast, UK). (ii) An Olympus IX-81 (Tokyo, Japan) inverted microscope in objective-type TIR configuration was used for imaging via an Olympus 60× NA = 1.49 Plan-Apochromat objective. Excitation of GFP was performed with the 488 nm emission of a diode laser (Toptica Photonics, Munich, Germany). Ninety-six-well plates were placed on an x-y-stage (CMR-STG-MHIX2-motorized table). Scanning of larger areas was supported by a laser-guided automated focus-hold system (ZDC2, Nikon, Tokyo, Japan). After appropriate filtering, the fluorescence signal was recorded via an Orca EM-CCD camera (Hamamatsu Photonics, Herrsching, Germany).

### 4.4. Prism-Type TIR Multiwell Reader

Cell seeding and treatment conditions were identical to those for objective-type TIRF imaging. A fluorescence multiwell reader for simultaneous TIR illumination of up to 96 samples in a microtiter plate with a custom-made glass bottom has been described previously [26]. For this study, the same experimental setup was used with a few modifications. Ninety-six-well microtiter plates without a bottom (Greiner Bio-One, Frickenhausen, Germany) were fixed to a Borofloat glass bottom of 2 mm thickness and low intrinsic luminescence by a non-cytotoxic silicon adhesive. This glass bottom permitted transmission of laser light and irradiation of all wells under an angle of 66° and thus above the critical angle of total internal reflection. A supercontinuum fiber laser (SuperK, NKT Photonics, Birkeröd, Denmark) emitting high repetition pulses (78 MHz) of short duration (5 ps) in a broad spectral range (430 nm–2.4 µm) was used for excitation of GFP, selecting a wavelength range of 450–490 nm by a SuperK VARIA Single Line Filter (NKT Photonics). The experimental setup was used for continuous wave (cw) fluorescence measurements in combination with a long-pass filter for λ ≥ 515 nm and a wide-angle objective lens (f’ = 25 mm) placed in front of a video camera (Axiocam HRm operated with software Axiovision 3.1, Carl Zeiss, Jena, Germany). Thus, the whole microtiter plate could be imaged simultaneously; however, in view of homogeneous illumination and aberration-free imaging, quantitative evaluation was restricted to 8 × 5 = 40 central wells (see Figure S2C).

### 4.5. Fluorescence Spectra for Autofluorescence and Quenching Properties of Plant Extracts

Plant extracts were diluted in KRPH buffer to 1, 10, and 25 mg/L and transferred to 96-well microtiter plates. A partial fluorescence spectrum was recorded with a microplate reader (Infinite M200, Tecan Group, Männedorf, Switzerland), using excitations from 450 to 530 nm in 10 nm steps and emission wavelengths 35 nm higher, respectively. The same procedure was performed with additional 0.5 mg/L GFP to detect potential quenching effects by the extracts.

### 4.6. Giant Plasma Membrane Vesicle (GPMV) Formation and Confocal Imaging

CHO-K1 hIR/GLUT4-myc-GFP cells were grown and treated the same way as for objective-type TIRF imaging with the following modifications. The cells were incubated with the test substances for 10 min, and then GPMV formation was initiated. After the cells were washed three times with GPMV buffer, they were incubated in GPMV-forming buffer for 1 h. All incubation steps were performed in a humidified atmosphere (≥95%) at 37 °C and 5% CO_2_. Finally, the buffer with formed GPMVs was transferred into empty wells of a 96-well imaging plate. The imaging was performed on the abovementioned Olympus IX-81 inverted microscope equipped with an IX2-DSU confocal unit. A light guide-coupled illumination system (Olympus U-HGLGPS, Tokyo, Japan) with appropriate filters (FITC) was used to image GFP fluorescence. The fluorescence signal was recorded by an Orca EM-CCD camera (Hamamatsu Photonics, Herrsching, Germany).

### 4.7. Data Analysis

Initial imaging recordings of objective-type TIRF microscopy were supported by the Nikon NIS Elements (version 4.4, Tokyo, Japan) or the Olympus Xcellence RT (version 2.1, Tokyo, Japan) software. In-depth analysis for the calculation of the fluorescence intensity in individual cells and a fast comparison of the fluorescent signal in numerous cells at different time intervals was performed using the Spotty framework. Each image recording from the automated large-area scan was processed as the following: all cells mapped on a single image were chosen by drawing respective ROIs. Calculated mean intensity values were corrected by fluorescence background for each image (ROI drawn in an area without any cells). Spotty can be retrieved online at https://bioinformatics.fh-hagenberg.at/site/fileadmin/user_upload/img_upload/projects/spotty.html. GPMV images were analyzed with in-house software implemented in MATLAB R2018a (MathWorks, Natick, MA, USA). Hence, fluorescence intensity counts per area of the GPMV membrane were detected by selection of the outer ring of the vesicles. The data sets were cleaned from outliers using the Hampel algorithm. This method uses the absolute deviation as a robust statistical value for the estimation of outliers [47]. TIR multiwell reader images were exported as TIFF files, and quantitation of fluorescence intensity over time was carried out in ImageJ (version 1.53, National Institute of Health, Bethesda, MD, USA) using the Multi-Measure plugin. Central ROIs in each illuminated well were chosen for mean fluorescence intensity calculation. Fluorescence background was subtracted for each well by measuring the mean intensity values for at least three wells for each multiwell plate without cells. Statistical analysis for all measurements was carried out using an unpaired *t*-test in GraphPad Prism (version 6.02, GraphPad Software Inc., San Diego, CA, USA).

## 5. Conclusions

In conclusion, a combination of the three discussed methods enables the quantitation of GLUT4 translocation with high throughput and high content. These results could improve and accelerate the identification and qualification of novel insulin mimetic compounds. A photophysical characterization of the tested compounds prior to cell-based fluorescence microscopy experiments appears reasonable.

## Supplementary Materials

Supplementary Materials can be found at https://www.mdpi.com/1422-0067/21/21/7964/s1.

## Abbreviations

GLUT4	Glucose transporter 4
GPMV	Giant plasma membrane vesicle
T2DM	Type 2 diabetes mellitus
TIRF	Total internal reflection fluorescence
