# Supplementary material for: Fluorescence Microscopy-Based Quantitation of GLUT4 Translocation: High Throughput or High Content?
Source: Int J Mol Sci. 2020 Oct 27;21(21):7964. doi: 10.3390/ijms21217964 (PMC7662403; doi:10.3390/ijms21217964)
Supplement: Supplementary file 1 [file ijms-21-07964-s001.pdf]

International Journal of
Molecular Sciences

MDPI

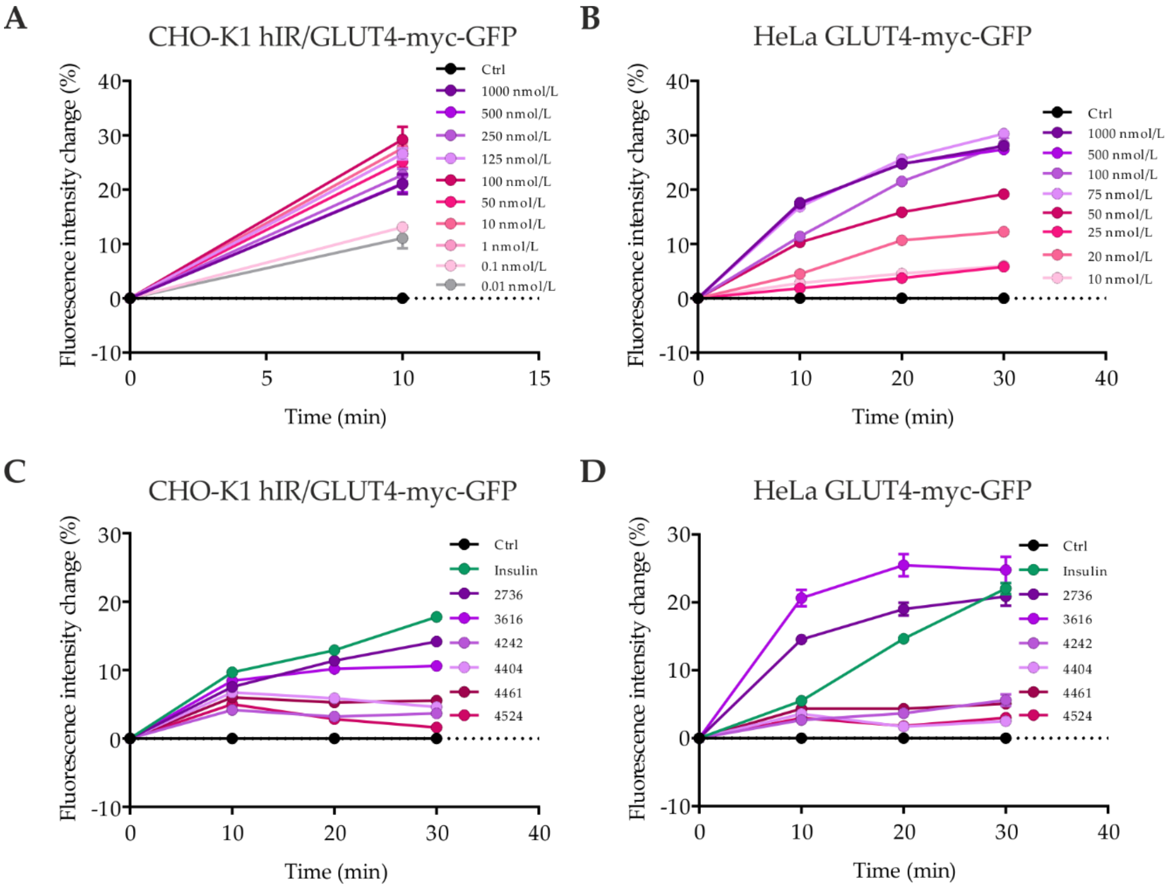

Figure S1. Temporal resolution of GLUT4 translocation quantitation by objective-type scanning TIRF microscopy. CHO-K1 (**A**, **C**) and HeLa (**B**, **D**) cells stably expressing a GLUT4-myc-GFP fusion protein were seeded into 96-well microtiter plates and starved for 3 h in HBSS buffer on the following day. Images in TIR configuration were taken before and after stimulation with insulin (A, B) or the indicated plant extracts (C, D) for 10, 20 and 30 min. Values represent mean ± SEM ($n > 500$).

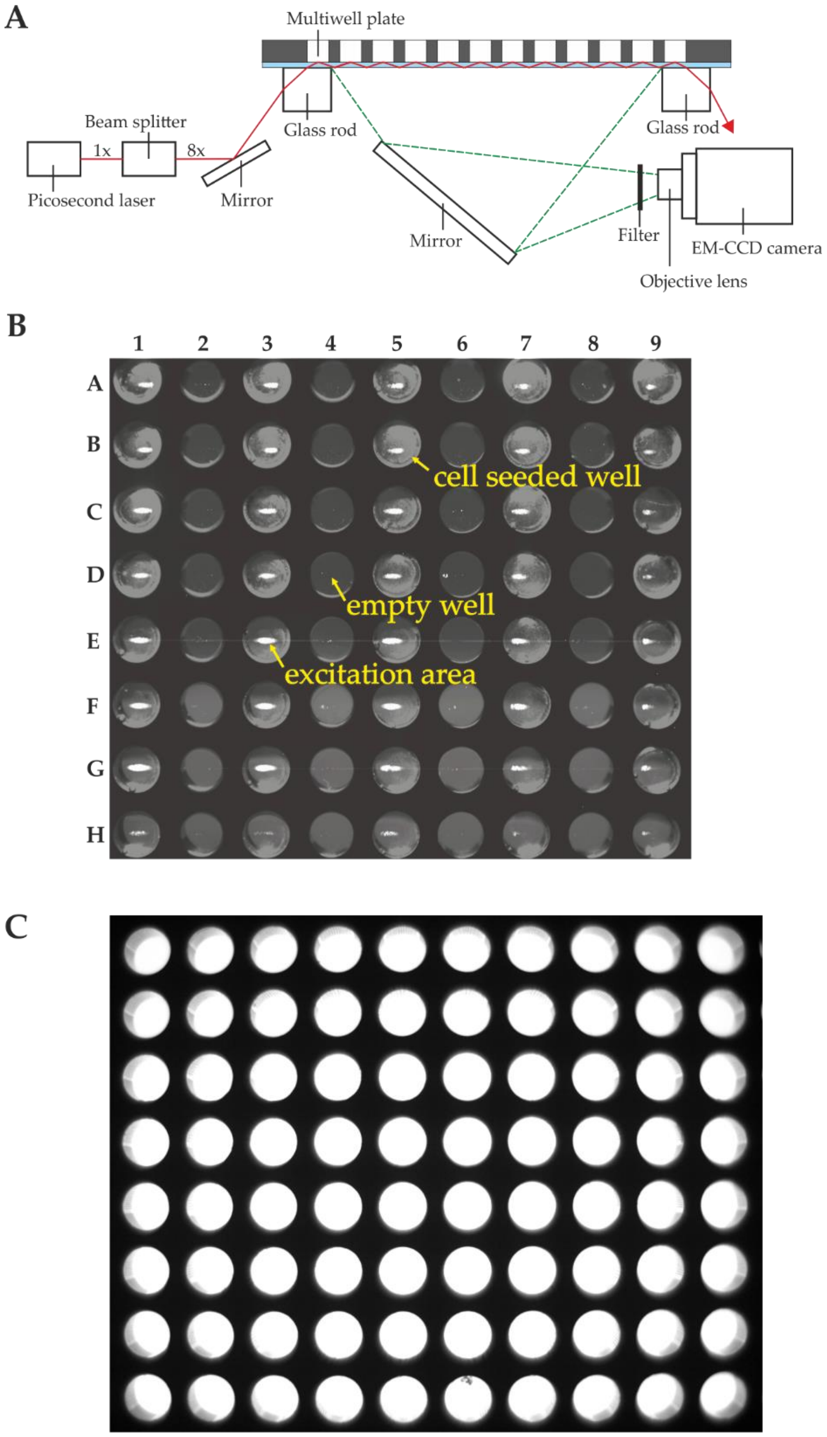

Figure S2. (**A**) Schematic illustration of prism-type TIR reader setup using 8x beam-splitting and 12-fold total internal reflection of each laser beam to illuminate 96 samples of a microtiter plate. (**B**) Representative image section of a mounted 96-well microtiter plate seeded with CHO-K1 cells stably expressing a GLUT4-myc-GFP fusion protein (column 1, 3, 5, 7, 9). (**C**) Illuminated microtiter plate with distortion and shading effects in wells at the edges.

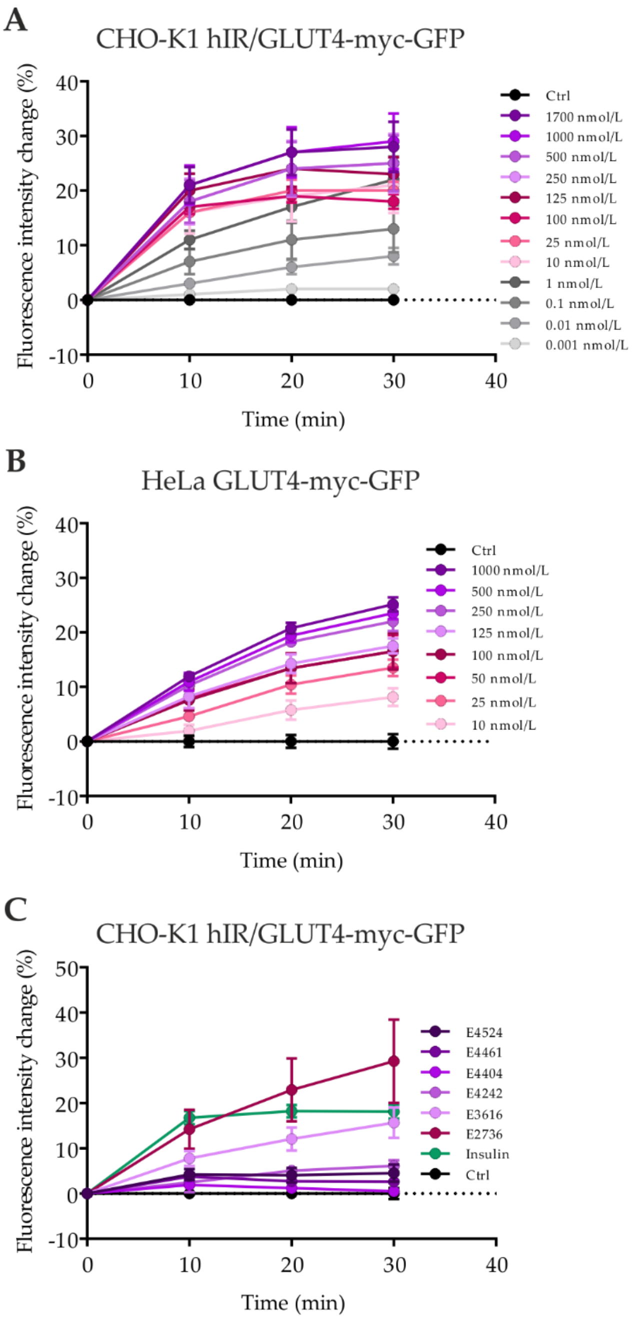

Figure S3. Temporal resolution of GLUT4 translocation quantitation using a prism-type TIR reader. CHO-K1 (**A**, **C**) and HeLa (**B**) cells stably expressing a GLUT4-myc-GFP fusion protein were seeded into specialized 96-well microtiter plates and starved for 3 h in HBSS buffer on the following day. Images in TIR configuration were taken before and after stimulation with insulin (A, B) or the indicated plant extracts (C) for 10, 20 and 30 min. Values represent mean ± SEM (n > 500).

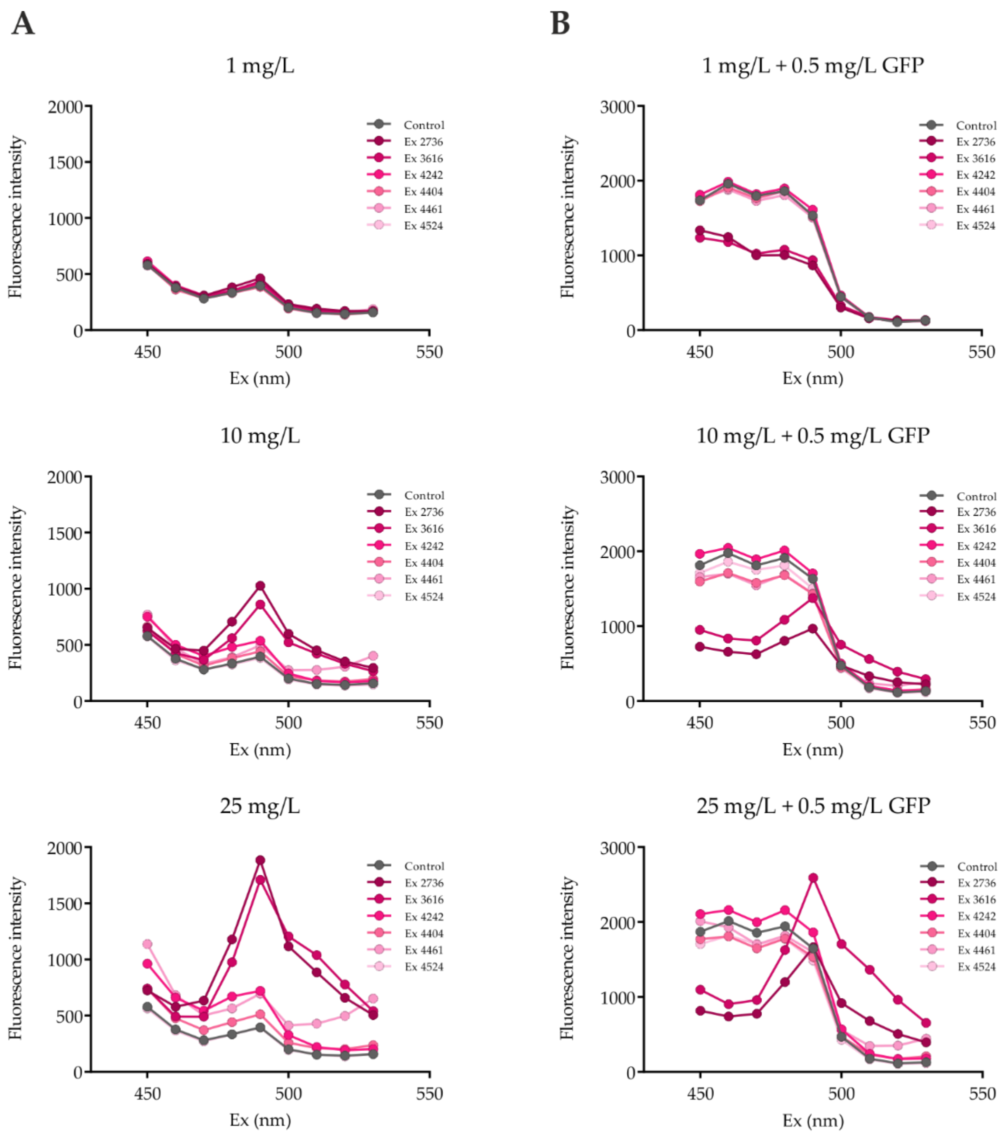

Figure S4. Fluorescence spectra of plant extracts for detection of autofluorescence and quenching properties. Fluorescence spectra of plant extracts in the concentrations 1, 10 and 25 mg/L were recorded in an excitation range from 450 to 530 nm (A). For analysis of quenching properties, the extracts were combined with 0.5 mg/L GFP and the same spectra were detected (B).
